# Comparing Social Responses To Ebola & Covid-19 In Sierra Leone: An Institutional Analysis

**DOI:** 10.1017/S002193202400021X

**Published:** 2024-05-16

**Authors:** Paul Richards, Foday Kamara, Esther Mokuwa, Marion Nyakoi

**Affiliations:** School of Environmental Studies, Njala University, Mokonde, Sierra Leone; Wellcome Pandemic Preparedness Study, Njala University, Mokonde, Sierra Leone; Development Economics Group, Wageningen University, Netherlands; Wellcome Pandemic Preparedness Study, Eastern Technical University, Kenema, Sierra Leone

## Abstract

This paper compares community responses to Ebola and Covid-19 in two regions of southern and eastern Sierra Leone with reference to the theory of institutional dynamics proposed by the anthropologist Mary Douglas. Institutions, Douglas argued, are conveyed by styles of thought, shaped by the ways in which human communities, through everyday practices, reinforce systems of classification and denotation. Pandemic advice to ‘follow the science’ proved problematic, since there is no single institution of science, and institutions never stand alone, but are bundled with other institutions, reflecting the manifold and intertwined practices of human social life. The paper explores some of the ways in which a traumatic epidemic of Ebola Virus Disease in Sierra Leone shaped a distinctive local response to deadly infectious disease in the absence of effective vaccine. This local approach emphasised social rules based on ideas about sequestration and testing. Communities then proposed to continue this rules-based approach to the pandemic of Covid-19 and showed little initial enthusiasm for vaccination. With Ebola, adoption of rules resulted in dramatic drops in infection rates. But Covid-19 spreads in different ways, and good results from the application of social rules were much less apparent. The paper shows how communities began to grapple with this new situation. In some cases, vaccine hesitation was overcome by treating the requirement for vaccine as a new form of social discipline. More generally, it is concluded that epidemiologists need to pay specific attention to institutions and institutional dynamics in order better to understand and anticipate public reactions to new disease threats.

## Introduction

Ebola Virus Disease is a viral haemorrhagic fever spread by contact with body fluids from an infected patient. Covid-19 is viral respiratory (lung) disease spread mainly by droplets and aerosols expelled during breathing and accelerated by coughing and sneezing by an infected person. Control of spread for Ebola involves patient isolation, barrier nursing and (now) vaccination. Control for Covid-19 infection involves social distancing, mask wearing, and (now) vaccination. An epidemic of Ebola occurred in 2013-15, involving inter-country spread mainly in Upper West Africa (Guinea, Liberia and Sierra Leone in particular). Covid-19 became a global pandemic during 2020.

The present study seeks to compare societal responses to Ebola and Covid-19 in Sierra Leone. To do so it draws on the theory of institutions. Institutions (according to Mary Douglas) are built out of actions and experiences and become ‘thought styles’ (sets of unthinkingly applied rules and reactions) which regulate responses among members of a group, without the group being consciously aware of the regulatory work being done (Douglas 1986, 1992, 1995, Richards & 6 2023). Douglas earlier tried to capture her notion of ‘how institutions think’ with words like ‘collective representation’ and ‘cosmology’, but later decided to adopt ‘thought style’, a term first suggested by the Polish scientist Ludwik Fleck in an analysis of how communities of researchers within medical science shaped research priorities (Douglas 1986: 12-14).

According to Douglas, institutionalization is driven by practical and symbolic (ritualised) social action and interaction and is reinforced by exemplification and natural analogy. An institution then imposes classification on people and events. Importantly, the work of institutions includes classification of risks. Classification invariably results in anomaly – bits that do not fit a given institutional scheme. Anomalies are resolved through various procedures – reclassification, exploitation, rejection, or (in some cases) simply by ignoring them (Douglas 1966, ch. 2, Richards & 6 2023, ch. 6).

Experience of infection is universal and is everywhere subjected to the above-enumerated processes of institutionalization. The social and ritual actions involved in attending to infection will shape and support a ‘thought style’ of infection management. These thought styles vary from place to place, however. This is due to three circumstances. Not all communities have been exposed to the same sets of diseases, infection processes are often misrecognised or disputed (especially when there are intervening vectors, as in the case of insect-transmitted diseases such as malaria or Yellow Fever, Barcia 2020), and institutions never stand alone, but belong to a tightly intertwined bundle of other institutions which are mutually supporting and have been shaped by local social and historical experience.

In an epidemic, or pandemic, the infectious process is engaged locally in a variety of different ways, depending on the specificities of the local institutional bundle. In some settings institutionalised professional medicine plays an important part, though tightly bound up with other societal institutions for security and social welfare. In other places there is less institutionalised professional medicine, and epidemic response, social welfare and even security may devolve largely upon institutions of family and community.

It is important to take account of the notion that part of the functionality of an institution is an autopilot element. People do what they do because they accept their institutions without question. Specifically, they are blinded to the way their own familiar institutional are bundled, reinforced by natural analogies and intensely applied exemplification. This means that appeals to rational adjustment are often ineffective. Too much is at stake in trying to tease apart mutually supporting institutions for adjustment or reform. The autopilot element guarantees a degree of social solidarity essential to survive hardship, but it also has down-sides – the tendency to believe self-fulfilling prophecies, for example.

When epidemiologists are surprised by the ways in which the public responds to an epidemic (whether positively or negatively) it should be asked whether this is the result of institutionalization (or deinstitutionalization). Analysing processes of institutionalization and deinstitutionalization of epidemic disease is a major explanatory task for the social sciences. Comparative data are hard to acquire but, where available, these can offer useful pointers towards solving institutional puzzles. It is the path pursued in this paper when comparing institutionalised responses to Ebola Virus Disease and Covid-19.

The case study country, Sierra Leone, relies a great deal on family mutual assistance in addressing challenges of ill-health, and this an advantage in a poor country where only a limited amount of professional medical assistance can be offered. But it also means that it was hard to persuade people that they should seek specialised help in nursing and burying those infected with the Ebola virus. On the other hand, following local rules for controlling spread of Ebola by limiting movement of people (both quarantine and self-sequestration), worked well, and played a major part in the downturn of the epidemic. A question addressed in this paper is whether this community-level engagement with Ebola Virus Disease left behind an institutional legacy appropriate to engagement with pandemic of Covid-19, and what adjustment was then required.

### Pandemic preparedness: an ethnographic approach

A study of pandemic preparedness was funded in two countries, Sierra Leone and Uganda, based on an anthropological approach. The present paper sums up some of the main findings of the Sierra Leone research team, based on ethnographic observation in two communities in central and eastern Sierra Leone over a period from September 2019 to August 2022. An aim of the study was to understand how communities conceptualised and responded to health threats including infectious diseases, and thus to begin to envisage how these communities might respond to a potential pandemic.

The research design involved selection of a community (in eastern Sierra Leone) badly affected by the outbreak of Ebola virus disease in 2014-15, and a second community (in central Sierra Leone) in which infection had been avoided. Understanding infection issues within the wider context of daily life involved participant observation, including regular conversations about health topics and social life more generally with a broad cross section of members of each community. At first, the pandemic challenge was hypothetical in these community discussions. Not long into the first year of fieldwork, however, the challenge changed, with the arrival of the first cases of Covid-19 in Sierra Leone in April 2020. Henceforth, enquiries were focused mainly on a comparison of Ebola and Covid-19, and on topics arising such as quarantine and vaccination.

This paper begins with an ethnographic example of how ideas about infection are embedded within specific institutional contexts, to bring out clearly what an anthropological approach to epidemiology comprises, and why an analysis from an institutional perspective adds value. The paper then explores a comparison between Ebola and Covid-19, bringing out (in particular) how community responses diverged.

#### Incest and infection

Mende is the main language spoken in our two case-study communities. According to the *Mende-English Dictionary* (Innes 1969) the word *simongama* (definite singular form) translates as ‘incest’. Sex between brothers and sisters, or with an animal would be considered *simongama*, and special steps have to be taken to protect the community against harm should a case be uncovered. As is typical in the defence of institutions, people will readily give a reason to explain why a rule is needed. They will often insist that incest is against nature. As Douglas (1986) explains, natural analogy is protective of institutions, but the institution is, in fact, social. This can be seen clearly when Mende people explain that co-habitation with a son or daughter of the father’s brother (the parallel cousin) would be considered *simongama*, but that co-habitation with the son or daughter of the mother’s brother (the cross cousin) is permitted, and indeed encouraged.

The institution in question, of permitting cross-cousin marriage but forbidding marriage with parallel cousins, is widely distributed among the peoples of coastal Upper West Africa. It appears to function to reinforce local cooperation. In a survey of one of the settlements of the cluster of central Sierra Leonean communities where fieldwork was based, 17 of 98 marriages were of this form, but nearly all (15/98) were to be found in a section of the community known as *tawoveihun* (old town), suggesting it was an institution particularly favoured by and protective of the interests of the longest-settled families.

In October 2019, the village of G. – the largest settlement among a cluster of research communities in central Sierra Leone – was alarmed by a case of *simongama*. A teenage schoolboy, paying a visit to his relative, a leader of village Muslims, needed to relieve himself at night, and afraid to go to the bush, found a convenient spot in a part-constructed building, but disturbed a tethered goat. Hearing the cries of the goat, the caretaker of the animal rushed across to confront the boy, who then fled, abandoning some of his items. He was later confronted with his belongings, which he acknowledged were his. Meanwhile, some women who had examined the goat reported that its reproductive organs were damaged. This was enough to lead to an accusation of *simongama*, which the boy vehemently denied. It transpired the goat was sick and had suffered a recent miscarriage. The caretaker of the animal may have been looking for a pretext to explain the goat’s poor state of health to its owner, but meanwhile it became widely believed that the boy had been trying to have sexual relations with the animal. Community alarm was aroused, and chiefs became involved in the case. The Imam, responsible for the boy, agreed to settle the case by paying small fine, not because he believed the charges were true, but because as a spiritual leader he was expected to be on the side of morality. *Simongama* was sin, and likely to put the community at risk.

A summary of the incident presented below is based on fieldnotes over the period October-November 2019 recorded by Foday Mahmoud Kamara, who observed the case and interviewed the key participants:

*MS [female] tells us that such an act creates fear in in the minds of people about eating goats in the community. That is because people fear eating humans indirectly. It will reduce their numbers in the village and spread sicknesses through flies that sit on the affected goat. it also stains the chiefdom [when people hear of] humans that have sex with animals*.

*MM [female] suggested this is not the first time the accused boy has been doing it. She noted that many goats and sheep have been dying, which is very unusual in the community. The act is totally wrong. The boy should be taken for thorough examination*.

*FM [female] maintained the act was treated lightly because of family connection [between the chief and Imam], and that in the long run this would adversely affect the community. The act was totally wrong. Thinking about the health implication on the community, the boy and the chiefdom at large, the chiefs judging the matter were not being fair with the people. The boy should have been isolated for a couple of days and examined before allowing him to mingle with others. By moving up and down he will affect others in the village*.

*SB [male] argued that the boy should be taken to community health unit or even or hospital for all possible tests to know whether he contracted any infection from the goat. His wife then chipped in, supporting the idea that the boy should be taken for thorough examination. SB added that they are still confused by the way the boy continues to deny the act. To avoid stain and build confidence in people, thorough treatment should be given to him so that he will not infect others*.

G has a health post staffed by nurses. The nurses were also asked for their views on the case:

*Nurse F thought that such an act may have a long-term effect on the boy, as no one can predict his health status, and the illness that killed the goat is not known. To start with, the human system is quite different from lower animals; the infection might be in his* [boy or goat?] *system for a long period of time undetected. Family members and the community should take the boy for various tests to allay the fears of the public*.

*Nurse R reflected on the after-effects for the boy and community at large. She thought that all tests should be administered to ensure that the boy is healthy and proves negative from infection. She added that if tests are not done, the community will be at risk for any possible outbreak, saying that these are the ways infections get transmitted to others*.

*The Imam gave his point of view. One evening he saw large crowed approaching his house with A. [the accused boy] stripped naked and crying. People said they had exhibits* [sic.] *relating to A. that testify that he had sex with a she goat in an unfinished house. When chased, [the boy] ran away and only later appeared. They showed him the exhibits and A. admitted that he is the owner of the items. But still he argues that he went to the house to ease himself. After A. undressed S. rushed on him pointing a torch. He had no alternative but to run away from the window leaving his items behind. He explained he normally went to the field but that particular night decided to toilet in the house because it was night*.

*The Imam also stated that S. slapped the boy for having sex with his goat and when asked to produce the animal confirmed that it was his goat. After some time, the boy complained of a serious headache. When asked to treat the boy for causing the condition S. did not answer. The Imam then summonsed S. to the chiefs’ [court] but he later settled the case with an apology. With all this, the boy still denied the act. Even when they told him they are going to swear him* [a customary oath-taking ceremony], *he said ‘no problem’*.

*The matter was then taken to the chiefs where the boy’s host finally said he is going to take the responsibility because of his status as the Chief Imam, and that people highly regard him, and expect much from him. He was fined Le 500, 000 and appealed the chiefs to reduce it to Le 100, 000 and pardon the boy. He ended his appeal by saying that he will send the boy for test*.

*The Animal Health Worker stationed in G, who also doubles as the sanitary officer, was asked for his view. He condemned the act but said he was not informed. For a man to have sex with a lower animal is wrong for the simple fact that the blood streams are not the same. If anyone does that it is making people eat human meat indirectly. The after-effect can be enormous and stain the community or the chiefdom at large. He then reflected on his work as an animal health worker. He works in the interest of the community. Many illnesses are contracted through animals if they are not properly controlled. He said even their droppings and urine can transmit illnesses if stepped on with bare feet. He said that is why people are advised to build housing for animals and confine them to these pens. They should not be left on free range. Despite all the advice, people still leave them on free range*.

*Chief JL, who handled the matter [in court], gave the following account. When he heard the news, he felt embarrassed. However, he said that until they have heard the case they cannot pass final judgement. The matter has been adjourned. Since it is a chiefdom crime, one or two chiefs alone cannot decide on it, except the Paramount Chief and other elders of the chiefdom are involved. The matter is still pending*.

*Chief JL later updated his response, saying that they have almost pardoned the boy, based on the fact that his father is the Imam and has accepted, because of his status, that his boy is guilty. But his boy denied the alleged act. He also reported that the boy said even if they come with any medicine he will swear* [on it – i.e. strongly deny].

*Chief JL was asked whether the test they might apply is traditional or medical. He clarified that he meant a medical test in the hospital, to actually prove the boy’s innocence. Asked further how they will know* [be able to identify] *if the boy had sex with the goat he stated that after the test result proves positive with infection it will then be concluded that the boy had sex with the goat*.

Chief JL was then asked to rate the gravity of the case:

*He suggested that it is not too serious, and for that reason they did not inform the Paramount Chief, though other people told him about it. The Paramount Chief came to G. while they were still dealing with case but did not ask about it. Chief JL said that the reason they did not explain the case* [to the Paramount Chief] *was because the boy’s father had shown a sign of respect* [to the sub-chiefs] *and pleaded by* [paying a fine of] *one hundred thousand Leones. As far as the chiefs are concerned, the matter has been laid to rest*.

#### Infection as an institution

A first point to stress in interpreting these remarks is that institutions never act alone. Here we see clearly how ideas about disease and infection are tightly bound up with ideas about sexual activity and how, when correctly applied, such activities play an essential part in constituting communities, but that when incorrectly applied they threaten social order. The most obvious threat is that infection will be fostered as a punishment for poor behaviour.

Infection, however, works in social as well as physiological ways, e.g. through the threat that eating the goat would constitute an indirect form of cannibalism, the most severe form of moral delinquency in the eyes of many Mende villagers (Richards 2020).

A second point is that facts are readily ignored when community institutions are in danger. As Douglas suggests, institutions make us blind to inconvenient facts. None of the informants appears to have shown much interest in thinking through the implications of the goat already being in poor health, due to a miscarriage, even though this information appears to have been widely known. Instead, the institutional ‘bundle’ for health in G. encouraged large and implausible leaps – such as assuming it was proof the boy was guilty because many sheep and goats had recently died, something it was asserted did not happen before. This is notwithstanding local spread of *peste des petits ruminants* (PPR), a viral disease of goats and sheep associated with high animal death rates, spread nationally by re-stocking programmes after the civil war (1991-2002). PPR was sufficiently well-known in rural communities by 2014 for it to be used in the Ebola epidemic as a ‘model’ for explaining the Ebola threat (Richards 2016: 108, 158).

Third, it is important to note the evidence of how the institution of chieftaincy works in rural Sierra Leone. British colonial rule was extended to the interior of Sierra - henceforth known as the Protectorate – in 1896. Threatened with a house tax they felt too poor to pay, the Protectorate chiefs rose against the British in 1898. The uprising was put down with military force, and the British then invested in a railway to bring the Protectorate closer to Freetown rule. Local institutions were coopted under a system of indirect administration – chiefs were made responsible for maintaining local order in return for certain privileges. The system was continued at independence as the lowest tier of local administration, even though the prestige of chiefs was damaged by the intrusion of party politics into their appointment.

Post-colonial decay of the chieftaincy system was deemed by many to be a factor in the 11-year brutal civil war (1991-2002). The Blair government sent the UK army to Sierra Leone to help restore peace, and then (at the request of an elected national government) agreed to fund the rehabilitation of the chieftaincy system. Chiefs were rehoused and given training and a code of conduct. Maintenance of security was one of their first tasks. This included monitoring for risks such as disease outbreaks.

As the above materials suggest, chiefs have two masters – they are responsible not only to the national government but also to local public opinion. That opinion polices its institutional boundaries with some rigour – *simongama* is not a threat to be taken lightly:

*The act was being treated lightly because of family connection, and in the long run this would adversely affect the community*. [FM, female, farmer, G, 7 November 2019]

Maybe the chiefs took into account the sickness and miscarriage of the goat in concluding that the case was not important enough to be escalated to the level of the Paramount Chief. But they do not put it this way. Chief JL is clear that the case was resolved by the Imam accepting the boy’s guilt (even though hedged with caveats) and through showing respect to the minor chiefs by paying a modest fine. This aspect of the case is important because (as will be shown below) actions by chiefs were crucial to Ebola control. But it needs to be stressed that chieftaincy, as an institution, has a logic of its own, and interlocks with other local institutions. It thereby engages with ideas about infection and quarantine on its own terms, and not those that might be presupposed by a public health specialist. Thus, it is important to understand, in Douglas’s terms, ‘how an institution thinks’ (Douglas 1986), and not to make guesses about what might or ought to happen based on the institutionalization of professional medicine.

This leads us on to a fourth point, highly significant for the theme of pandemic preparedness, concerning how, locally, people absorb science into their local institutional thinking. Rapid testing was crucial to Ebola control. The disease presents in two phases – three days of ‘dry’ symptoms (fever, severe headache and so forth) largely indistinguishable from malaria, the most widespread disease in the country, and a second phase of ‘wet’ symptoms (again of about three days, leading to organ failure and death, or recovery). Patient handling is relatively risk-free during the dry phase, but very risky (in terms of infection of the nurse or carer) in the ‘wet’ phase. Patients and families were very unwilling to agree to isolation in a case handling facility due to risk of cross-infection with Ebola while the case remained ‘dry’, and it was still possible the cause was malaria, not Ebola. The key to effective control of the epidemic, therefore, was rapid testing of blood samples, to confirm Ebola and isolate the patient before the case turned ‘wet’.

Accordingly, responders invested heavily in testing (including three mobile laboratories supplied by the Dutch government) to ensure rapid turn-round of samples. The population at large became highly conscious of testing, which affected many more than had the disease. The phrase ‘I know my status’ – meaning ‘I have tested negative for Ebola’ - quickly passed into popular parlance as an everyday guarantee that people could continue to interact socially.

We are dealing here with the part of institutionalization that Douglas terms ‘exemplification’ (Douglas 1992, Richards & 6 2023). A negative test result exemplifies that a person is safe to be re-admitted to the community. Some of this experience is reflected in information supplied above. Chief JL clarified that he meant a medical test in the hospital to prove the boy’s innocence by proving he was free from any kind of infection.

*Asked how they will know* [be able to identify] *if the boy had sex with the goat Chief JL answered that after the test result proves positive with infection it will then be concluded that the boy had sex with the goat*.

Doubtless boosted by learning from Ebola, local institutionalization had absorbed a message about the existence and importance of hospital testing, but not the technical details – that tests need to be for something specific, and that no link has yet been shown by science between *simongama* and specific infectious diseases.

Nurse R was clear that testing is powerfully protective of the community (a lesson learned from Ebola) but was unspecific as to what test should be applied:

*All tests should be administered to ensure that the boy is healthy and proves negative from infection. If tests are not done, the community will be at risk for any possible outbreak; these are the ways infections get transmitted to others*. [Nurse R, health centre, village G]

The boy’s guardian, the Imam, was unconvinced the boy was guilty as charged, but nevertheless agreed to plead guilty on his behalf and pay a fine, to close the case. The risk of reputational damage was far too high to fight over details. It is worth noting that several informants pointed out this reputational damage is significant at a chiefdom as well as a personal level. As will be further discussed below, chiefdoms competed in terms of their record at reducing cases of Ebola; people thought it important that their chiefdom showed to the world its citizens were serious about restricting this deadly disease. The Imam agreed to send the boy for testing for similar reasons, but for precisely what we never discovered. Post-Ebola in Sierra Leone, testing has an institutionalized force as exemplification way beyond its practical capacity. In institutionalized forms of thinking, hope and need often far outstrip technical means.

### Comparing Ebola and Covid-19 – institutionally

#### Ebola – a disease that is terrifyingly self-evident

Most infections with Ebola result from nursing a ‘wet’ case, or from preparing the corpse for burial. Islam is the majority world faith in Sierra Leone and most families expect to follow Islamic rules on corpse preparation, which include washing the body and expelling waste material, before dressing the corpse in a white shroud (Mende, *kasange*). ‘Wet’ cases require specialized handling. These procedures include barrier nursing and ‘safe’ interment (burial in a plastic body bag, without washing of the corpse).

The first Ebola case-handling facilities (from June 2014) were improvised and poorly equipped. Staff struggled heroically in nearly impossible conditions, and some centres became little more than death traps, none more so than a case handling centre serving the area of our central Sierra Leonean case study (Richards et al. 2020). It seems possible that a number of suspected cases in this centre were, in fact, admitted with malaria, but then died after becoming cross-infected with Ebola due to slow testing and poor patient separation.

Mid-epidemic (September 2014 onwards), Red Cross, MSF and other agencies built several large case handling facilities – Ebola Treatment Centres (ETC) - to a high design standard, and secured fleets of ambulances to move patients in safer conditions. The earliest treatment protocols in ETC excluded rehydration via an intravenous line, except in the latest stages of the disease. The army 34 Military Hospital in Hastings experimented with early line rehydration, and halved death-rates (interview with General [Professor] Foday Sahr, E. Y. Mokuwa, personal communication). Subsequently, this became the preferred procedure, and now families had an incentive to allow patients to be transferred to ETC; their chances of survival were higher.

Even so, many families delayed seeking help. Memories of earlier experiences with chaotic case handling were hard to erase. The families preferred to gamble the case was no more than malaria or typhoid, until wet symptoms proved otherwise, by which time moving the patient was highly hazardous to all concerned.

Community Care Centres (from November 2014) proved to be a game changer (Mokuwa & Maat 2020). These were small facilities (of 10-16 beds) built out of sticks and canvas and rapidly erected in places beginning to experience the onset of cases. Staffed by ‘volunteers’ (newly qualified nurses without government appointments) and community recruits for non-medical jobs such as cleaning and cooking, the CCC were close to the families of new cases, made rapid testing a priority, and treated and discharged all Ebola-negative cases. Families could regularly check progress, talk to patients through a simple chain fence and bring home-prepared food as requested. Functioning more like village general purpose clinics than ETC, CCC provided families with every encouragement to bring patients promptly for testing, and this greatly improved case detection and isolation rates, helping bring down case numbers in newly infected areas. This drove a local discourse comparing CCC and the established Peripheral Health Units (Mokuwa & Richards 2022) with CCC often being seen as superior. This contributed to the local institutionalization of some aspects of infection control under Ebola (notably the focus on testing, a major demand in the ethnographic case described above).

There was an equivalent transformation of ‘safe burial’. Safe burial practice was initially organized by development agencies, who found it easier to recruit and train burial teams in towns. The urban ‘hired help’ became notorious for its ritual insensitivity (they were reported to dump bodies in graves with the assistance of poles, rather gently laying corpses in the correct aspect as Muslim tradition requires), and change was persistently demanded by affected communities (Richards 2016).

The argument was always the same. If you have money to train these people, then come here and train us - we will do the job as well, but with sensitivity towards the dead and sympathy towards the living (Richards 2016). There were some models for locally based burial teams, because in Kailahun, where the epidemic first surged without a fully coordinated government or international response, communities had had to cope on their own. Our eastern case study was of a village where there had been 89 cases of Ebola and 69 deaths, starting in the first weeks of the epidemic in May 2014. The Paramount Chief, who lost his own wife and daughter as early victims of this outbreak, was a former health worker, and used his contacts rapidly to inform himself on the rules of Ebola infection control. He then implemented key developments including, crucially, the deployment of a chiefdom youth volunteer force to undertake case finding, enforcement of movement restrictions, quarantine, and safe burial (Richards 2016: 126-32). Despite lack of equipment and supplies, such as rubber gloves and chlorine, these teams were remarkably free of infection, and their actions rapidly and visibly brought down infection numbers.

Having created a local model for effective Ebola control, this was then translated into sets of byelaws implemented by chiefdom councils, using the court disciplinary processes apparent in the ethnographic case described above. These byelaws were adopted widely in Kenema and Kailahun District, and finally endorsed by Parliament and rolled out nationally on 5^th^ August 2014. Cases had already begun to turn down in eastern districts before the international response became fully engaged from September-October 2014. The daily ‘dashboard’ of cases published by the National Ebola Response Commission (NERC) referred to the areas where infection chains had been ended as ‘silent districts’.

A degree of local institutional re-alignment then took place. People began to understand that in the absence of effective treatment or vaccination against Ebola, social discipline was a key factor in infection control. Disregarding, as entirely lacking in evidence, the favoured international explanation that Ebola was spread by eating bush meat, people on the ground extensively reported that the true cause of spread was human movement, and that the best antidote was self-sequestration (preventing visitors of ‘unknown status’ from entering communities, wherever possible). This work required renewed vigilance by chiefs, since it was long-established chiefdom law that no ‘stranger’ should be invited to stay overnight in a village without the chief being notified.

Local administrations also competed to turn their chiefdom from ‘red’ (active cases) to ‘green’ (no cases), even if, given the political salience of ‘red’ and ‘green’ as the colours associated with the two main rival political parties in Sierra Leone, the choice of colour coding might have been queried as somewhat unfortunate. Kamara et al. (2022) describe the strength of protest when one chiefdom Ebola task force committee found it had wrongly been credited with a ‘positive’ case (a local resident who had been infected, and recovered, elsewhere). The Paramount Chief took considerable trouble to have this anomaly re-classified (exactly as Douglas [1986] predicts). The minutes of the chiefdom Ebola task force committee record that if the anomaly could not be removed administratively then the person should be obliged to remove themselves physically (Kamara et al. 2022)!

In all, people concluded that the way to beat Ebola was to follow the law. What made this especially potent was that the laws had not been imposed from above. Experience had already shown that imposed laws met with resistance. Everyone knew the byelaws had been generated ‘from below’ in and through daily practices of preventing Ebola from spreading. The means of infection control was institutionally embedded within the Ebola affected communities. It happened that some of the chiefs who had codified these practices also had a degree of medical knowledge, so the local institution of infection control was kept in alignment with external advice on Ebola mitigation. There was no clash of institutions.

Where there was a potential misfit concerned women’s control over burial procedures, via the rituals of the Sande Society (a powerful women’s association found throughout rural Sierra Leone). Mokuwa (2020) has described how she helped resolve some potential disquiet on this issue, by exploring with society elders the extent to which they already possessed institutionalized knowledge of relevant infection control, acquired from dealing with earlier risks, such as those posed by smallpox, where isolation of patients in farms was practiced:

*My understanding on how people used to treat the sick* [in these cases] *is that anyone seriously sick will be taken to a particular section in the bush, and an announcement is made to the community that someone is suffering from a difficult illness and must be taken out of the community to be treated by an individual in the bush with native herbs. The sick person is taken out of the community for the illness not to spread in the community and affect others*. [Female, societal head, village G]

In summary, then, Ebola control was effective in Sierra Leone because there was a degree of alignment with relevant locally-institutionalised infection control procedures. With Covid-19 we shall find that the situation was different.

#### Covid-19 – a disease that is almost impossible to recognise

Covid-19 is a disease that spreads on the air, via droplets and aerosols. Most infection takes place indoors in poorly ventilated places. Rural Sierra Leoneans live much of their lives in the open air or buildings open to the breeze and protected only from the rain. Covid-19 mainly seriously affects older people, and Sierra Leone has a population pyramid with large numbers of youth. Additionally, there is some evidence that the worst sufferers from the disease are those who lack exercise (Ezzatvar et al. 2022). Many rural Sierra Leoneans do not attend gyms but work hard physically on farms and in kitchens and yards, and trek long distances on foot, to save the fare, even where motorcycle taxis are available, so fitness may have inhibited Covid-19.

For these reasons cases in rural areas are especially hard to spot, especially given that symptoms are close to those of several other respiratory complaints. At first, estimates of exposure to the disease are not very reliable. As one of the world’s poorest countries, Sierra Leone had only been able to organize limited population-level testing to sample undiagnosed cases. But a cross-sectional, nationally representative and age-stratified seroprevalence survey was then carried out in March 2021 and suggested that actual levels of exposure were 43 times higher than reported numbers of cases, even though ‘overall seroprevalence was low compared with countries in Europe and the Americas (suggesting relatively successful containment in Sierra Leone)’ (Barrie et al. 2022).

The World Health Organization also included the country in an attempt to model estimates of ‘excess death’ - the number of actual deaths minus the number there would have been in the absence of Covid-19 (WHO 2022). The calculation involves many assumptions, so caution is needed in interpretation. For example, the computation needs to take account of indirect deaths caused by the pandemic itself, including those who died because there no hospital staff due to sickness, or because quarantine closed markets and people were highly malnourished. But it is worth noting that overall, tropical African countries typically had only about half the average level of ‘excess death’ experienced in Europe and North America during the first two years of the pandemic, and Sierra Leone sits somewhere in the middle of the range of values for West Africa (Table 1). Much of this might be attributable to the large percentage of young people in the population. But it is also worth noting that ‘excess death’ increased in 2021 (a year when vaccines were available) over the average for 2020, a year before vaccines became available. This suggests some slackening of pressure to maintain social distancing and quarantine (the sets of rules that had also previously reduced Ebola cases). Many Sierra Leoneans believe they escaped Covid-19 by following the rules.

On the other hand, sufferers from the disease do not always recognize when they have been infected. We encountered a case in Bo in which a woman had been dispatched to an isolation ward in Freetown for a week, after twice testing positive for Covid-19, but when she was followed up on her return to Bo, vehemently denied she had suffered any symptoms, and claimed that as far as she could see the same was true of all the people on the Covid-19 ward with her. In her eyes, Covid-19 was a ‘fake’ disease, perhaps because she had been led to believe that it would have the dramatic impact of Ebola. Symptomless carriers, or persons with very mild infections, have been a major complicating factor in attempting to mitigate Covid-19.

Visibility was not an issue with Ebola. The terrifying signature of this disease was inescapable. There is some evidence that people exposed at home to Ebola cases may have developed immunities without ever having developed symptoms, but it is thought unlikely that symptomless carriers contributed much to immunity (Glynn et al. 2017).

Overwhelmingly, infection was passed on by ‘wet’ victims, and there was no doubting they were very ill indeed. There were, initially, some Ebola denialists, but this denial tended to wither on exposure to an actual case. As one informant told us, with Ebola ‘seeing was believing’. This was not the case with Covid-19. In fact, despite asking widely, we hardly encountered a person who could confidently say they had seen an actual case of Covid-19, or that they knew for certain that they had been infected. In the case of Covid-19 not seeing was not believing.

*I have never seen a* [Covid] *patient with my naked eyes. After the quarantine of early cases, I have not heard any new case in this country*. [FB, farmer, village G]

*Well for some, existence of the pandemic was not real, simply because they did not see a patient*. [ES, Women’s Leader, G]

This was a big problem for vaccination campaigns, especially when rumours spread that some vaccines might be dangerous.

*Many say they are not sure of the disease and have not set eyes on the patient. Above all, they are not sick, so they don’t need vaccine*. [AG, section chief, G]

A typical response was then to reject the need for vaccination: ‘why should I risk taking a vaccine when I am not ill’.

Nevertheless, vaccination was not entirely strange. An elderly woman farmer from village G. provided us with the following account of older curing practices and a subsequent innovation among rural Mende people, the introduction of vaccination known (in Krio) as *maklet* (the word derives from an obscure English word ‘maculate’ [Fyle & Jones 1980] meaning to spot or mark, mainly used by botanists):

*All illness was* [in those days] *handled with native medicine/herbs. There was no clinic or hospital. People were mainly getting healing from herbs. Since there was no hospital, my only resource was water and ginger. And to many people, ginger, white clay (wugei) and water were very good in curing many diseases. People are still using it today. Combination of boiled herbs served as the main source of cure for any illness; there was nothing like vaccination or clinic. I* [first] *noticed maklet* [vaccination] *when I was young. If my memory can serve me well* [*maklet*] *started during the time of Sir Milton Margai. It was a machine that was punched on us. You can even see the big black spot [scar] on my left arm*. [MS, elderly female farmer, G]

Yet doubts about vaccination persisted. Many villagers told us they had several objections to vaccinations. First, was the disease real? Second, the vaccines had been developed far faster than predicted, and were rumoured to be dangerous. Third, and perhaps most significantly, better treatments for the pandemic were already available, because Ebola had taught the importance of following rules on epidemics:

*It is a law and will lead to prevention from infection. This was what helped us in the days of Ebola. We should still stick to the laws so that it will end smoothly. But if all the laws are abandoned it will not end quickly* [JL, sub-chief, G]

*To stick to the laws, use of mask, avoid handshakes. We were so smart to arrest the* [Covid-19] *pandemic with early preparation, which is why it does not spread like wildfire*. [JK, female farmer, G]

In effect, and as seen in the incest case above, the first requirement of public health is a demand for chiefs to apply the law. From this institutionalised perspective Covid-19 would be ended by doubling down on laws relating to masking, social distancing and quarantine:

*To tell people to continuously use the mask. But our town chief does not seem to be serious in enforcing the law. Even the* [hand] *wash facilities given to the community, most are not making use of it. As authorities, we expect them to monitor and ensure that people adhere to all Covid-19 rules and regulations*. [FB, male farmer, G]

Attitudes to masking and lockdown varied by location and occupation. In the first period of the pandemic, before vaccines were available, we ran a small survey on masking, and what people thought about it, with a sample of villagers in G, and a group of people sampled in a working-class suburb of Bo. Most respondents in the first sample (equally divided between males and females) were farmers, who produced and stored food by collaborating at household and inter-household level. Many respondents in the second sample (again equally divided by gender) were petty traders, who fed from what they earned each day, in competition with rivals. We asked people whether they wore a mask and (for those that said ‘yes’) why they wore it. It was striking that about half the sample of farmers explained they wore a mask to protect others. Nearly all the traders – if they wore a mask at all (many said they could not afford them) – mentioned only that the mask protected them from infection. This is a clear instance of two distinct processes of institutionalization stressing the importance of cooperation among small farmers and the importance of competition among small traders.

Others reported that lockdown rules were too damaging: *Our paramount chief instituted byelaws to regulate the behaviour of people*: *we were deprived of our self, movement and business* [JD, woman trader, G]

There was also some outright defiance of lockdown rules, which closed local markets in the countryside, but (oddly) not in town. The rural reaction was at times vehement. Women in G complained they had lost all their trading capital. In NG, nursing mothers struggled to find milk for children and were reduced to making very imperfect substitutes with rice pap and a little sugar if they could still find any. People then started taking the law into their own hands. Farmers risked action by army or police to return clandestinely to their farms. A periodic market at M was re-opened by the traders before the government lifted lockdown. The market people were prepared to fight the authorities, since people had run out of necessities. Fortunately, the government realised lockdown was counter-productive and controls were promptly rescinded.

Some aspects of Ebola response are also remembered in highly negative terms, notably the large Ebola Treatment Centres and the often panic-inspired indiscriminate spraying of chlorine, which in the popular imagination (at least) was responsible for many unnecessary deaths. The laying of these complaints was sometimes accompanied by quite specific demands, for example for greater accountability on how the chlorine dilution was done.

*I have a fear of* [vaccination] *because of what happened during Ebola. Most were saying that when an Ebola patient goes for medication he or she will never come again. That is why I’m afraid; if they say, again, vaccine has come for Covid-19 I will fear to take it*. [MS, elderly female farmer, G]

*Many people died* [in the Ebola epidemic] *because of chlorine sprayed on them. Many people even blame members of the burial team for finally killing their relatives. When one is sick of asthma and taken to the ambulance, the first aid was chlorine, which added more suffering to the patients. Some of them still remember this bitter experience* [HB, farmer, G]

### Conclusion: following the science?

To end pandemic Covid-19 countries are advised to ‘follow the science’. Villagers in Mende-speaking rural communities in Sierra Leone have come to a different conclusion. Many think that Ebola was reduced by following rules on quarantine, social distancing and burial. These rules were developed and applied locally, boosting confidence in local institutions of law and order. The defeat of Ebola was a success for rule-following behaviour and explains why the epidemic was in retreat in the parts of Sierra Leone first infected with the disease even before the international Ebola response arrived (Richards 2016, ch. 5).

Papkalla and Schroven (2016: 10) report that workshops to assess lessons learned from the Ebola epidemic in Sierra Leone ‘underlined …the power of community by-laws [sic.] as an instrument for behaviour and structural change’. But as this paper has argued, rule-following behaviour was much less effective when applied to Covid-19, because of the much lower visibility of the disease. To be effective, rules must be seen to work. There was little scope to measure the effectiveness of rules as a check on Covid-19 when the disease is so hard to identify. Many of the rules were self-evidently disregarded. Not wearing masks undermined the resolve of others. Economic lockdowns quickly produced life-threatening hardship (e.g. disruptions to basic food supplies) and had to be promptly abandoned. The rules-based approach to management of Covid-19 lost traction as a result.

If Sierra Leone’s record of ‘excess death’ from Covid-19 is modest by comparison with countries like Russia or the United States (Table 1) it is unlikely to have been a result of rule-following behaviour since rules were often quickly revoked. But would ‘following the science’ prove to be a better alternative? The problem with this advice is it fails to take account of institutions, and how institutions guide human thought. Science is itself an institution, with many local and disciplinary institutional variants. But it is, everywhere, only one set of institutions among many. Even in the United Kingdom, which considers itself to be a science-oriented country, battles continue to rage over institutional interactions – the scientific advisory committee approach to lockdown, as opposed to lockdown policy as viewed by the Exchequer, for example. So, it makes no sense to advise ‘follow the science’ without first assessing the extent to which this advice is the product of a set of institutions, bundled with other sets of institutions, each bundle configured differently for its own region and history.

Science has long been active within Sierra Leone, and recently effective in tracking Covid-19 (Barrie et al. 2022). As we have tried to show, the history of science in Sierra Leone is perhaps surprisingly apparent within village discourse, for example over *maklet* [vaccination]. This suggests that with relevant ethnographic and historical insights it may be possible to enlarge the discourse of science within such settings. The failure of the rules-based approach to cope with the challenge of Covid-19 thus represents something of an opportunity. Indeed, some of the village sceptics we interviewed presented very clear arguments about why vaccination (the preferred scientific answer to Covid-19) was anomalous both on its own institutional terms (for example, in terms of speed of development and release) and in local institutional terms (in terms of the expectation that vaccine was something given to children [Mokuwa 2024]). They then openly issued an appeal – let the government come to us to explain the challenges posed by this virus and how vaccination works to solve these challenges. Others were even more explicit, suggesting that the problem of trust in medicine would only be solved by local manufacture of medicines and vaccines.

In other words, the road to institutional development along scientific lines is open in Africa, but only if science takes full account of the problem of how institutions think, and how that thinking in turn shapes the outcomes of science.

## Figures and Tables

**Table 1:** EXCESS DEATHS PER 100,000 (World Health Organization model data for Covid-19)

WEST AFRICA	2020	2021	Ave. both years
Benin	32	64	48
Burkina Faso	34	70	52
Cameroon	46	85	66
Cote d’Ivoire	30	65	48
Gambia	41	77	59
Ghana	21	46	33
**Guinea**	30	64	47
Guinea Bissau	56	86	71
**Liberia**	27	52	39
Mali	62	71	66
Mauretania	64	83	74
Niger	66	73	70
Nigeria	29	60	45
Senegal	29	68	49
**Sierra Leone**	45	53	49
Togo	-63	-21	-42
**OTHER AFRICAN COUNTRIES**			
South Africa	92	307	200
Uganda	6	38	22
**OTHER TROPICAL COUNTRIES**			
Costa Rica	27	161	94
Haiti	25	58	42
Panama	69	106	88
**OTHER COUNTRIES**			
USA	141	140	140
France	75	50	63
Germany	80	153	116
Ireland	9	50	29
Italy	166	100	133
Netherlands	85	86	85
Sweden	84	27	56
UK	126	93	109
New Zealand	-43	-13	-28
Israel	28	43	35
Russian Federation	253	482	367

## EXPLANATORY NOTE

Excess death is a number computed to reflect the additional deaths caused by Covid-19 over all other factors causing death. The computation includes deaths caused by indirect effects of pandemic, such as lockdown or lack of medical treatment. A negative value implies that Covid-19 (or exogenous factors linked to the pandemic) may have reduced other causes of death. For instance, strong social distancing may protect from Covid-19 infection and other respiratory infections. Computing excess death is not a straightforward matter and involves assumptions (https://www.who.int/publications/m/item/methods-for-estimating-the-excess-mortality-associatedwith-the-covid-19-pandemic). Provided the assumptions are accepted excess death figures can be used to compare pandemic responses in different countries. The WHO results cited here from running an excess death model relate to two years of the pandemic (2020 and 2021). Table 1 lists figures for West Africa, and some other countries, in those two years. The country with highest excess death, worldwide, is the Russian Federation. The countries with the lowest figures are Togo and New Zealand. In the West African data 2021 is worse than 2020, probably reflecting slow vaccine roll-out. The difference between the two years is least in the case of Sierra Leone, though Sierra Leone was in the middle of the range for excess death in 2020. Overall, Ghana has the lowest average of excess deaths over both years, and Mauretania the highest. Excess death was middle of the regional range for three Ebola-affected countries - Guinea, Liberia and Sierra Leone. West African countries are lower in terms of estimated ‘excess deaths’ than South Africa, but not so low as Uganda. On a pan-tropical comparison, West Africa does better than central American tropical countries such as Costa Rica and Panama. When the comparison is with Europe, ‘excess death’ rates for France, Sweden and Ireland are equivalent to rates in West Africa, but UK, Germany, Italy and the Netherlands have higher rates than West Africa. In the US, vaccine rollout appears to have had little effect on ‘excess death’, perhaps due to high levels of vaccine refusal in some areas. Despite lack of vaccine access, West African excess death does not appear high by global standards, perhaps resulting from the youthfulness of the population. It is to be noted that rates doubled in 2021 (a year when vaccines became available) compared to 2020 when countries were dependent only on ‘social’ methods of infection control (such as byelaws and social distancing).
